# Intrinsically Disordered Side of the Zika Virus Proteome

**DOI:** 10.3389/fcimb.2016.00144

**Published:** 2016-11-04

**Authors:** Rajanish Giri, Deepak Kumar, Nitin Sharma, Vladimir N. Uversky

**Affiliations:** ^1^School of Basic Sciences, Indian Institute of Technology MandiMandi, India; ^2^Department of Molecular Medicine and Byrd Alzheimer's Research Institute, Morsani College of Medicine, University of South FloridaTampa, FL, USA; ^3^Laboratory of Structural Dynamics, Stability and Folding of Proteins, Institute of Cytology, Russian Academy of SciencesSaint Petersburg, Russia

**Keywords:** Zika virus, intrinsically disordered proteins (IDPs), intrinsically disordered protein regions (IDPRs), molecular machinery, viral proteome, cellular proteome

## Abstract

Over the last few decades, concepts of protein intrinsic disorder have been implicated in different biological processes. Recent studies have suggested that intrinsically disordered proteins (IDPs) provide structural plasticity and functional diversity to viral proteins that are involved in rapid replication and immune evasion in host cells. In case of Zika virus, the roles of protein intrinsic disorder in mechanisms of pathogenesis are not completely understood. In this study, we have analyzed the prevalence of intrinsic disorder in Zika virus proteome (strain MR 766). Our analyses revealed that Zika virus polyprotein is enriched with intrinsically disordered protein regions (IDPRs) and this finding is consistent with previous reports on the involvement of IDPs in shell formation and virulence of the *Flaviviridae* family. We found abundant IDPRs in Capsid, NS2B, NS3, NS4A, and NS5 proteins that are involved in mature particle formation and replication. In our view, the intrinsic disorder-focused analysis of ZIKV proteins could be important for the development of disorder-based drugs.

## Introduction

In 1947, Zika virus (ZIKV) was first identified in Uganda through a monitoring network of sylvatic yellow fever in rhesus monkeys (Dick et al., 1952). Outbreaks of ZIKV-related disease have been recorded throughout southern Africa with a high number of birth defects and abnormalities including microcephaly, intracranial calcification, and fetal death (Petersen et al., 2016). As many other members the *Flaviviridae* family, ZIKV is an arbovirus transmitted through the infected arthropods (by the bites of the infected mosquitoes from the *Aedes* genus, *Ae. aegypti* and *Ae. albopictus*). Therefore, distribution of Zika infection is mainly associated with the distribution of *Aedes* mosquito vectors that can be found in different parts of the world (Wikan and Smith, 2016). World Health Organization (WHO) has declared Zika related problems as public health emergency of international concern. Therefore, it is of utmost urgency to find out mechanism of pathogenesis of this virus and to develop therapeutics. A recent study on immunocompetent mouse model has strengthened the previous observations that ZIKV infection might cause neurological defects in fetuses (Lazear et al., 2016). This virus is transmitted through mosquitos, as well as via blood transfusion and also from mother to fetus during pregnancy (Wikan and Smith, 2016). Reports also suggest the possibility of sexual transmission (Grischott et al., 2016).

**Figure 1:**
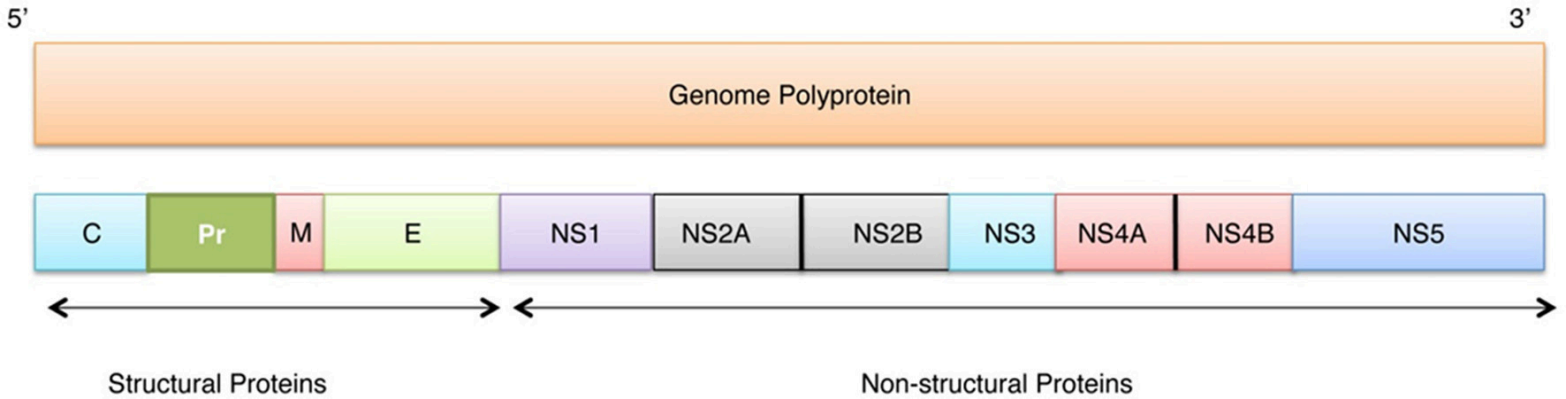
**Localization of individual proteins within the genome polyprotein of Zika virus (Q32ZE1)**. Top bar shows ZIKV RNA (10974 bases) that translates into polyprotein of 3418 residues (bottom bar) that at maturation is cleaved into three structural proteins (Capsid (C), Precursor membrane (prM), and Envelope protein (E)) and seven non-structural proteins (NS1, NS2A, NS2B, NS3, NS4A, NS4B, NS5).

ZIKV belongs to the *Flaviviridae* family, genus *Flavivirus*, which includes several important human pathogens, such as West Nile virus (WNV), Dengue virus (DENV), Yellow fever virus (YFV), and Japanese encephalitis virus (JEV). Recently, ZIKV structure has been solved by cryo-electron microscopy (Sirohi et al., 2016). Genome of this virus includes a single-stranded RNA consisting 10794 bases along with two non-coding regions known as the 5′ NCR and the 3′ NCR. The open reading frame (ORF) of the ZIKV, concerning the protein expression order is as follows: 5′-C-prM-E-NS1-NS2A-NS2B-NS3-NS4A-NS4B-NS5-3′. It codes for a single polyprotein that is posttranslationally cleaved into three structural proteins (Capsid (C), Precursor membrane (prM) protein, and Envelope (E) protein), and seven non-structural proteins (NS1, NS2A, NS2B, NS3, NS4A, NS4B, and NS5) (see Figure 1).

The major focus of this article is on intrinsic disorder-centered analysis of ZIKV proteome. In general, intrinsically disordered proteins (IDPs) are recently recognized class of proteins that lack stable three-dimensional structure in their native state but still functional. Structurally, these proteins are highly heterogeneous and include random coils, pre-molten globules, molten globules, proteins with large flexible linkers, and hybrid proteins containing ordered and disordered regions (Wright and Dyson, 2015). Lack of structure in IDPs and intrinsically disordered protein regions (IDPRs) allow the interaction of proteins with several partners, thereby regulating multiple signaling pathways. These multiple interactions are attributed to fast binding kinetics of IDPs/IDPRs that is regulated through coupled folding and binding mechanisms (Gianni et al., 2012). Disordered regions provide greater capture radii that increase the probability to interact with partners. This mechanism is known as the fly casting mechanism (Shoemaker et al., 2000). Commonly, viruses have highly compact genome and contain disordered protein regions. This could be one of the reasons for high mutagenic capacity of viruses (Xue et al., 2014). Recent studies have reported that core proteins of the viruses from the *Flaviviridae* family contain significant amount of IDPRs (Xue et al., 2014; Goh et al., 2016). A functional correlation between intrinsic disorder and protein function has been established in proteomes of other *Flaviviridae* family members, such as Dengue Virus (Meng et al., 2015) and Hepatitis C Virus (HCV) (Fan et al., 2014).

In our view, the intrinsic disorder-focused analysis of ZIKV proteins could be important for the development of new disorder-based drug strategies (Cheng et al., 2006; Uversky, 2010, 2012). Current drug development strategies have shown to utilize combination of conventional drug design and computational approaches to target dynamic ensembles of IDPs (Ambadipudi and Zweckstetter, 2016). In the current work, we have analyzed the penetrance of intrinsic disorder in the ZIKV proteome. Further, we have correlated the abundance of structural disorder with functionality of ZIKV proteins. This study provides a novel direction for elucidating the mechanism of virus-host interaction. Some proteins have been already used as drug targets in other flaviviruses, such as NS3 helicase, envelope glycoproteins, NS2B-NS3 (serine protease) and NS5 (RNA-directed RNA Polymerase) (Li et al., 2008; Poh et al., 2009; Mayhoub et al., 2011). Therefore, results of our current work should be considered before inhibitor designing.

## Materials and methods

Reviewed and experimentally validated polyprotein sequence (UniProt ID: Q32ZE1) of the Zika virus strain Mr766 was used for the disorder analysis. There are several protein intrinsic disorder predictors developed, such as multiple members of the PONDR® family [e.g., PONDR® FIT (Xue et al., 2010), PONDR® VLXT (Romero et al., 2001), and PONDR® VSL2 (Obradovic et al., 2005), IUPred (Dosztanyi et al., 2005a), GlobPlot (Linding et al., 2003b), DisoPred (Ward et al., 2004), SPRITZ (Vullo et al., 2006), DisEMBL (Linding et al., 2003a)], etc. Many of these predictors have been assessed for accuracy within the frames of the Critical Assessment of Protein Structure Prediction (CASP) (Deng et al., 2012). Since many of these predictors are considering phenomenon of intrinsic from different angles, it is advisable to use several computational tools while looking for the abundance of intrinsic disorder in query proteins. Therefore, PONDR® FIT (Xue et al., 2010), PONDR® VLXT (Romero et al., 2001), PONDR® VSL2 (Obradovic et al., 2005), and IUPred (Dosztanyi et al., 2005a) were used in our study for disorder prediction in polyprotein of ZIKV. We also extended our analysis over each individual protein derived from the ZIKV polyprotein. Here, scores above 0.5 are considered to correspond to the disordered residues/regions. PONDR® VSL2B is one of the more accurate stand-alone disorder predictors (Peng et al., 2005; Peng and Kurgan, 2012; Fan and Kurgan, 2014), PONDR® VLXT is known to have high sensitivity to local sequence peculiarities and can be used for identifying disorder-based interaction sites (Dunker et al., 2001), whereas a metapredictor PONDR-FIT is moderately more accurate than each of the component predictors (Xue et al., 2010), PONDR® VLXT (Dunker et al., 2001), PONDR® VSL2 (Peng et al., 2005), PONDR® VL3 (Peng et al., 2006), FoldIndex (Prilusky et al., 2005), IUPred (Dosztanyi et al., 2005a), TopIDP (Campen et al., 2008). IUPred was designed to recognize IDPRs from the amino acid sequence alone based on the estimated pairwise energy content, where it was hypothesized that globular proteins are composed of amino acids which have the potential to form a large number of favorable interactions, whereas IDPs/IDPRs do not have unique 3D structure because their amino acid composition does not allow sufficient favorable interactions to form (Dosztanyi et al., 2005a,b).

Often, IDPs/IDPRs are involved in protein-protein interactions and molecular recognitions (Dunker et al., 2002a,b, 2008; Tompa, 2002; Oldfield et al., 2005; Dunker and Uversky, 2008; Uversky and Dunker, 2010; Uversky, 2013b). There are numerous reports emphasizing that IDPs/IDPRs are able to undergo at least partial disorder-to-order transitions upon binding, which is crucial for recognition, regulation, and signaling. Among these potential functional sites are short order-prone motifs within long disordered regions that are able to undergo disorder-to-order transition during the binding to a specific partner. These motifs are known as molecular recognition feature (MoRF), and they can be identified computationally (Oldfield et al., 2005; Cheng et al., 2007). We used ANCHOR algorithm to identify potential disorder-based binding sites (Dosztanyi et al., 2009; Meszaros et al., 2009). This approach relies on the pairwise energy estimation approach developed for the general disorder prediction method IUPred (Dosztanyi et al., 2005a,b), being based on the hypothesis that long regions of disorder contain localized potential binding sites that cannot form enough favorable intrachain interactions to fold on their own, but are likely to gain stabilizing energy by interacting with a globular protein partner (Dosztanyi et al., 2009; Meszaros et al., 2009).

## Results and discussion

### Intrinsic disorder in ZIKV polyprotein

In crystallography-based protein structure characterization it is assumed that the disordered regions cannot crystalize, being present in the form of regions with missing electron density, and only ordered regions have the propensity for crystal formation (Uversky, 2013a). In addition to this, crystallization conditions frequently contain various additives (presence of PEG, high salt concentrations, etc.), which make these conditions to be different from the natural environment. Therefore, a computational analysis of disorder based on the amino acid sequence alone using various programs may provide a great advantage to analyze the disorder in proteins (Uversky, 2013a).

Despite being a big threat, the holistic understanding of ZIKV proteins in both ordered and disordered perspective has not been established as of yet. Despite obvious interest to Zika virus, crystallographic data are currently available only for four ZIKV proteins, NS1, NS2B-NS3 protease (residues 49 to 95 of NS2B covalently linked via Gly_4_-Ser-Gly_4_ to the N-terminal protease domain (residues 1 to 170) of NS3), NS3 (only Helicase domain), M and E proteins (see below).

**Figure 2:**
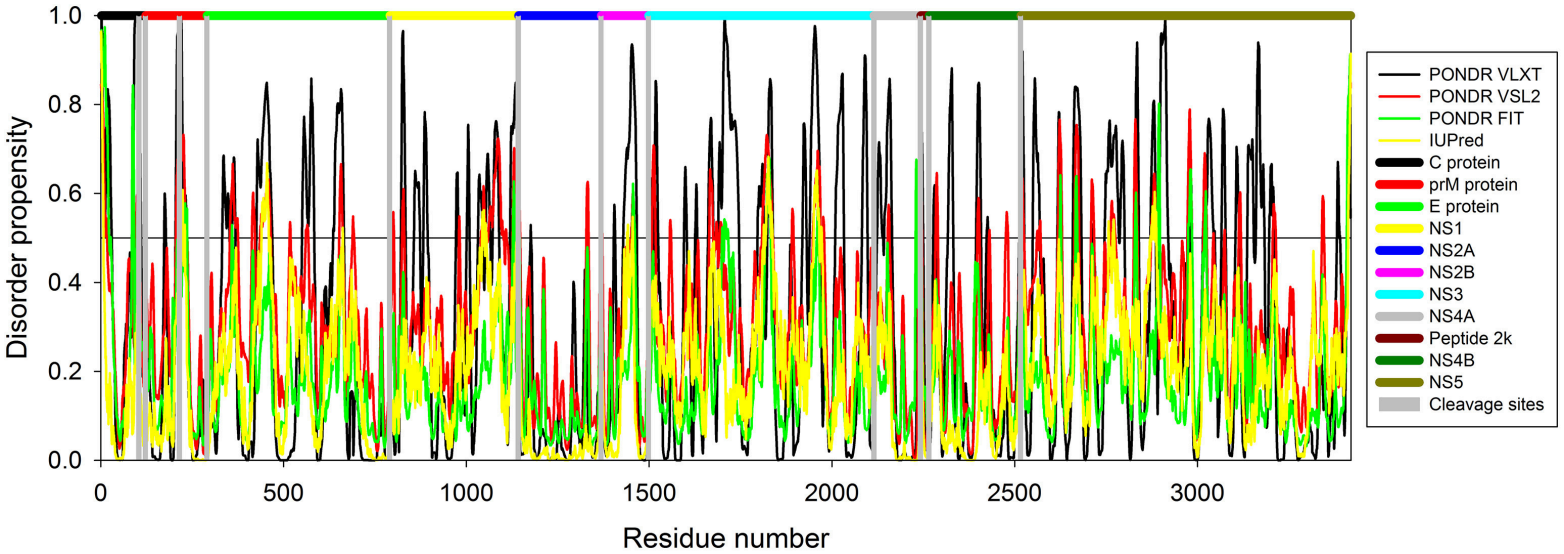
**Intrinsic disorder predisposition of Zika virus polyprotein (Q32ZE1)**. Disorder propensity is evaluated by PONDR® VLXT (black line), PONDR® VSL2 (red line), PONDR® FIT (green line) and IUPred (yellow line). Colored bars at the top of plot shows localization of the individual proteins. Corresponding cleavage sites leading for the generation of mature individual proteins are shown by gray bars. Disorder scores above the threshold 0.5 characterize residues/regions predicted to be disordered.

**Figure 3:**
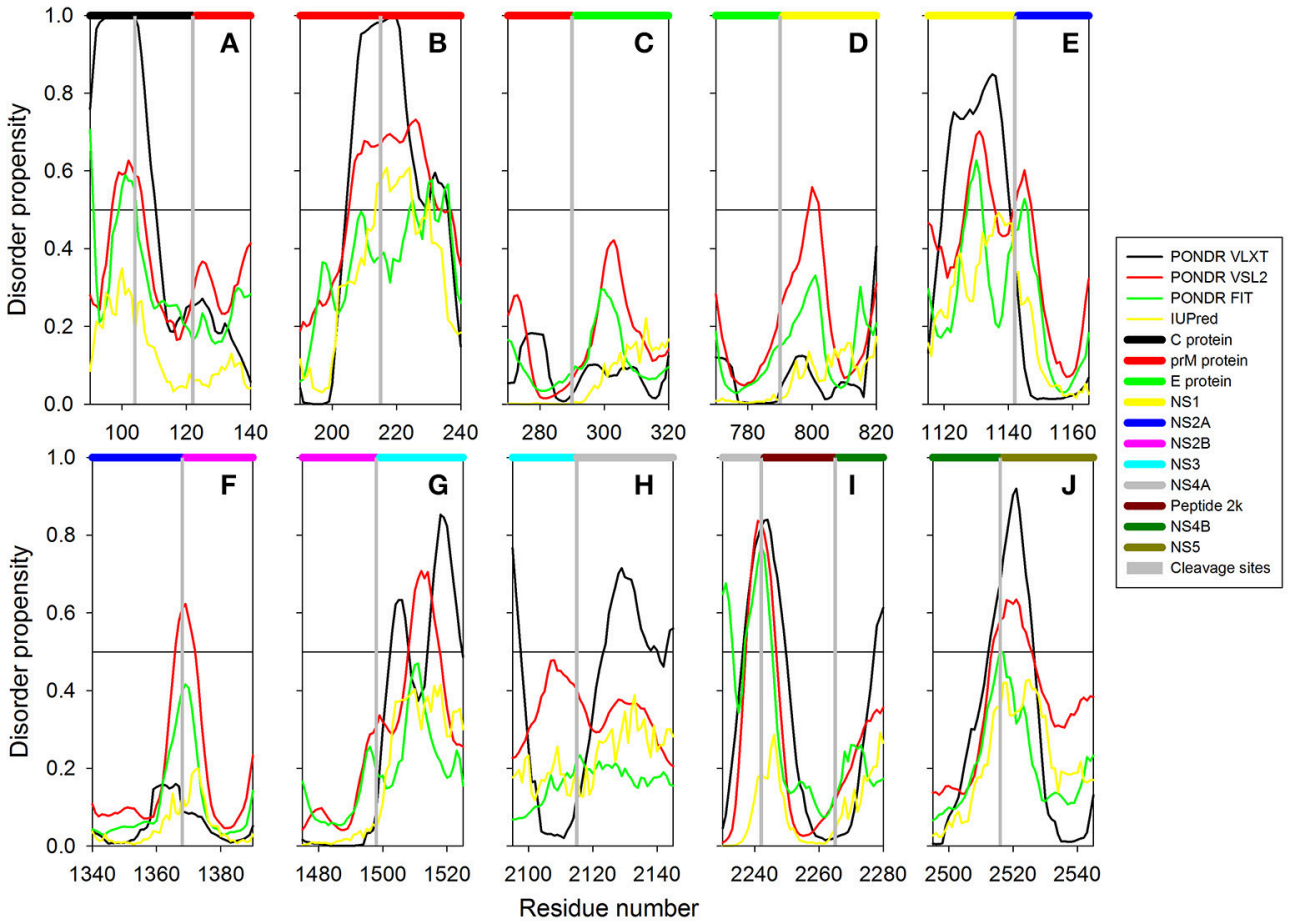
**The role of intrinsic disorder in maturation of individual proteins of Zika virus**. Plots shows position of cleavage sites (gray vertical bars) in relation to disorder profiles at the junction between the individual proteins within the polyprotein. **(A)** A cleavage site between proteins C (black horizontal bar) and prM (red horizontal bar). A cleavage site at the position 104 within the pro-protein C leading to the removal of propeptide (residues 105–122) is also shown. **(B)** A cleavage site within the prM protein leading to generation of proteins Pr and M. **(C)** A cleavage site between the proteins prM (red horizontal bar) and E (green horizontal bar). **(D)** A cleavage site between the proteins E (green horizontal bar) and NS1 (yellow horizontal bar). **(E)** A cleavage site between the proteins NS1 (yellow horizontal bar) and NS2A (blue horizontal bar). **(F)** A cleavage site between the proteins NS2A (blue horizontal bar) and NS2B (pink horizontal bar). **(G)** A cleavage site between the proteins NS2B (pink horizontal bar) and NS3 (cyan horizontal bar). **(H)** A cleavage site between the proteins NS3 (pink horizontal bar) and NS4A (gray horizontal bar). **(I)** Cleavage sites between the protein NS4A (gray horizontal bar) and the peptide 2k (dark red horizontal bar) and the peptide 2k (dark red horizontal bar) and protein NS4B (dark green horizontal bar). **(J)** A cleavage site between the proteins NS4B (dark green horizontal bar) and NS5 (dark yellow horizontal bar).

In this study we have computationally evaluated the predisposition of ZIKV polyprotein for intrinsic disorder (see Figure 2) and also studied intrinsic disorder propensity of all individual proteins derived from this polyprotein: Capsid protein C (residues 2-122, which includes protein C (residues 2-104) and the ER anchor for the protein C (residues 105-122), which is removed in mature form by serine protease NS3), precursor membrane protein prM (residues 123-290, which is further divided to peptide pr (residues 123-215) and small envelope protein M (residues 216-290)), an envelope protein E (residues 291-790), and seven non-structural proteins: NS1 (residues 791-1142), NS2A (residues 1143-1368), serine protease subunit NS2B (residues 1369-1498), serine protease NS3 (residues 1499-2115), NS4A (residues 2116-2242), peptide 2k (residues 2243-2265), NS4B (2266-2516), and RNA-directed RNA polymerase NS5 (residues 2517-3419). It is recognized that disordered regions provide structural flexibility leading to binding promiscuity often involved in cellular regulations (Xue et al., 2014; Wright and Dyson, 2015). Often, disordered or flexible regions contain sites of proteolytic cleavage, since proteolytic digestion is known to occur much faster in unstructured than in structured protein regions (Fontana et al., 1986, 2004; Novotny and Bruccoleri, 1987; Iakoucheva et al., 2001). This suggests that the sites of preferential cleavage should be preferentially located within the regions that lack stable structure or possess high structural flexibility. Earlier, this specific use of intrinsic disorder/flexibility for generation of mature viral proteins from the polyprotein was reported for two other representatives of the *Flaviviridae* family, HCV (Fan et al., 2014) and Dengue Virus (Meng et al., 2015). In agreement with these considerations, Figure 2 shows that, within the polyprotein, cleavage sites leading to the generation of mature ZIKV proteins are preferentially located within the disordered or flexible regions or at least in close proximity to the regions with increased flexibility. Figure 3 further zooms into this phenomenon and shows that in the vicinity of the vertical gray lines that correspond to the cleavage sites, the per-residue disorder propensity scores evaluated by at least one of the disorder predictors used in this study typically spike to relatively high values.

### Analysis of the IDPRs in ZIKV structural proteins

#### C protein

**Figure 4:**
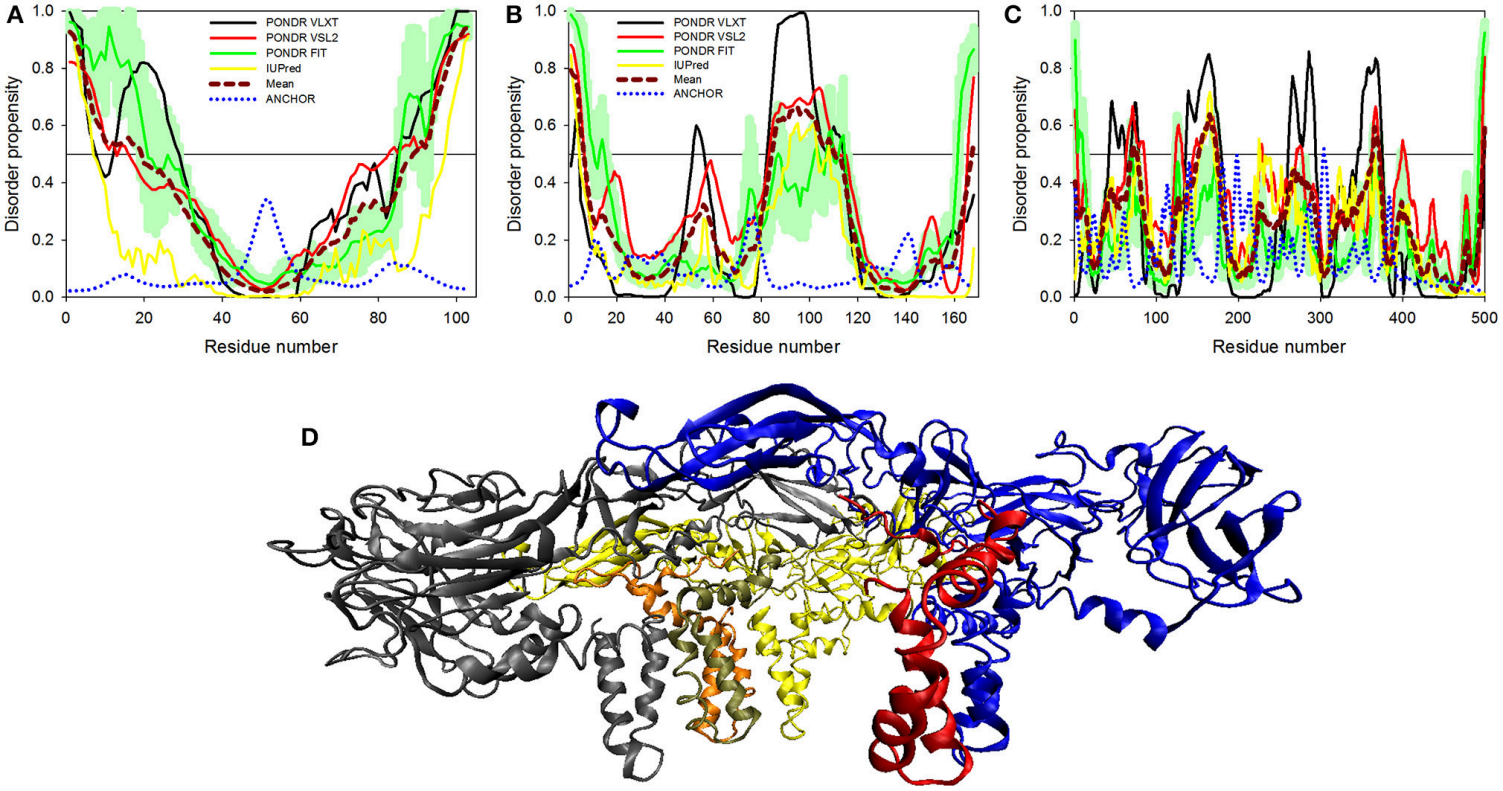
**Disorder predisposition of structural proteins of Zika virus. (A)** Capsid protein. **(B)** prM protein. **(C)** Protein E. Disorder profiles generated by PONDR® VLXT, PONDR® VSL2, PONDR® FIT, and IUPred are shown by black, red, green, and yellow lines, respectively. Dark red dashed line shows the mean disorder propensity calculated by averaging disorder profiles of individual predictors. Light green shadow around the PONDR® FIT shows error distribution. Blue dotted lines correspond to the result of the functional disorder analysis using ANCHOR algorithm. **(D)** 3D-structure of the trimer of E-M dimers (asymmetric unit) found in the ZIKV mature viral particle. This complex is formed between three E-M dimers colored as blue-red, gray-orange, and yellow-tan pairs, where structures of the E protein are shown in blue, gray and yellow, and where structures of the M protein are colored as red, orange and tan. The structure of the mature particle of Zika virus was determined by cryo-EM analysis (PDB ID: 5IRE) (Sirohi et al., 2016). Structural representation have been rendered using VMD (Humphrey et al., 1996).

Structural proteins in ZIKV consist of Capsid, prM and Envelope proteins. Capsid protein contains 102 residues and forms icosahedral capsid (30 nm in diameter) of the virus (Kuno and Chang, 2007), where the genomic RNA of ZIKV is encapsulated. Disordered regions of capsid protein include its N- and C-termini, with an overall predicted percent of intrinsic disorder (PPID) of 33.3% calculated from outputs of four predictors used in our study (Table 1 and Figure 4A). These observations are consistent with the results of previous studies on the disorder predisposition of proteins in flaviviruses (Goh et al., 2016). *In vitro* studies have implicated the role of intrinsic disorder for chaperone-like activities, such as viral genome packaging (Ivanyi-Nagy et al., 2008). In the case of Dengue virus, detailed functional study of capsid protein revealed that its N-terminal disordered region is responsible for carrying out multiple interactions necessary for mature virus particle formation (Martins et al., 2012). Furthermore, disordered N-terminal region interacts with phospholipids of lipid droplets (Martins et al., 2012). Similar studies have correlated the role of capsid disorder with the diverse functions in other flaviviruses, such as YFV and WNV. (Ivanyi-Nagy and Darlix, 2010) In other flaviviruses, it was shown that high virulence is correlated with the disorder levels of capsid protein (Goh et al., 2016). We assume that high abundance of disorder in capsid of ZIKV may provide an insight to uncover the mechanism of pathogenesis of this virus.

**Table 1:** **Some physicochemical and intrinsic disorder properties of Zika virus proteins**.

**Protein name**	**Length (M.W., kDa)**	**pI**	**PPID_VLXT_**	**PPID_VSL2_**	**PPID_FIT_**	**PPID_IUPred_**	**PPID_mean_**
C	103 (11.73)	12.0	40.8	34.0	36.9	12.6	33.3
prM	168 (19.01)	8.55	23.8	22.6	16.7	10.1	19.0
Pr	93 (10.51)	6.05	20.4	17.2	14.0	6.6	16.1
M	75 (8.51)	10.11	28.0	29.3	20.0	14.7	24.0
E	500 (54.09)	6.48	27.4	18.0	3.8	4.6	7.6
NS1	352 (40.08)	6.17	33.0	26.7	7.1	6.0	10.8
NS2A	226 (23.97)	10.34	7.5	7.5	9.7	3.1	5.7
NS2B	130 (13.77)	4.44	37.7	14.6	17.7	7.7	16.2
NS3	617 (68.41)	8.22	36.6	18.2	9.4	9.2	12.8
NS4A	127 (13.70)	5.82	40.2	12.6	23.6	6.3	16.5
NS4B	251 (26.94)	9.10	19.1	12.4	10.4	4.0	6.4
NS5	903 (103.02)	8.67	33.6	16.1	7.0	3.8	8.6

#### prM protein

Next to capsid is the protein known as prM that acts as a chaperone for envelope protein. prM protein shows a central role in transition of immature virus particle to mature form, which is infectious, virulent, fusogenic, and can adhere to host cell membrane (Zhang et al., 2003). Immature viral particles of flaviviruses are noninfectious and are characterized by their “spiky” shape, possessing 60 trimeric E-prM heterodimer spikes (Zhang et al., 2003). The mature viral particle is smooth and contains 90 dimeric E:M heterodimers (Kuhn et al., 2002; Zhang et al., 2013). The low-pH environment of the trans-Golgi network is crucial for the maturation of viral particles, since it leads to the conformational changes of the surface glycoproteins needed for the cleavage of prM by the host protease furin to generate the pr peptide and mature protein M (Kuno and Chang, 2007). Subsequent removal of the pr peptide leads to the exposure of the ~12–amino acid-long fusion loop on the E protein, which, in the immature virus, is protected by the pr peptide (Yu et al., 2008). With exposed fusion loop, the virus is prepared for the low pH–mediated endosomal fusion (Yu et al., 2008). Therefore, both proteins eventually generated from the prM are functionally important, where Pr peptide protects the fusion loop of the E protein, and M protein acts as a transmembrane protein in the mature viral particle (Yu et al., 2008).

ZIKV prM contains 168 amino acids. In addition to the disordered N-and C-termini there is a long disordered central region in prM located between residues 80 and 120. This region contains the furin cleavage site (see Figure 4B and Figure 3). A multitool computational disorder analysis shows that the overall disorder of prM is 19.0% (see Table 1). Since the proteolytic cleavage of prM generates two functionally important proteins, the pr peptide and M protein (Yu et al., 2008). The analysis of both proteins separately revealed the PPID of 16.1% and 24% for pr and M proteins respectively (see Table 1). Despite high level of predicted disorder, M protein is able to form stable structure, being complexed with the E protein (see Figure 4D). Structurally, ZIKV M protein is characterized by N-terminal soluble loop (M loop) which contain two short α-helices (residues 6–10 and 21–39) and two transmembrane α-helices (residues 40–52 and 56–71) connected by very short loop that forms the stem and the transmembrane part of this protein embedded in the lipid bilayer. (Liu et al., 2004; Sirohi et al., 2016). Curiously, although the soluble M loop is predicted to be mostly disordered and contains little regular secondary structure in the E-M complex, it is crucial for stabilization of the E-M dimer, being intercalated into the E protein structure. Therefore, it is likely that this region of the M protein undergoes functional disorder-to-order transition.

#### E protein

At the next step, we analyzed the envelope protein E (500 residues) that participates in the membrane fusion between host late endosomes and virion. Protein E heterodimerizes with M, and this complex stabilizes E protein likely due to the chaperone-like activity of M (Hamel et al., 2015). The averaged PPID of 7.6% is observed in E protein (Table 1), which is rather low. However, several IDPRs of different length are predicted in the ZIKV E protein (Figure 4C). Viral capsid consists of 180 copies each of the E glycoprotein and the M protein anchored in a lipid membrane (Sirohi et al., 2016). Structurally, E protein consists of four domains; stem transmembrane domain and three ectodomains t external surface of the viral capsid and are mostly β-structural (see Figure 4D).

Previous studies on other flaviviruses (e.g., Dengue virus and West Nile virus) demonstrated that glycosylation of the E protein at specific sites provides the ability to attach to different cell types (Beasley et al., 2005; Pokidysheva et al., 2006; Miller et al., 2008). Cryo-EM structure of mature ZIKV viral particle revealed that the glycosylation site is located at the loop region nearby the fusion peptide (Sirohi et al., 2016). Besides being needed for pH-mediated endosomal fusion, the ZIKV fusion loop (residues 98-110) can act as an epitope and react with different antibodies. Figure 4C shows that although this region is predicted to be mostly ordered, it has some weak tendency for the presence of disorder-based binding sites as evidenced by the output of the ANCHOR algorithm. In fact, the ANCHOR-based analysis of protein E revealed the absence of disordered binding regions that function via undergoing a disorder-to-order transition upon binding to a globular protein partner. However, the fusion loop is located in close proximity to the fifth highest spike found within the ANCHOR profile of protein E (see Figure 4C). Furthermore, our disorder analysis revealed that the longest disorder region found in ZIKV E protein (located within the 120-180 region) contains the glycosylation site (Asn154) (Sirohi et al., 2016). High disorder propensity of the loop encompassing the glycosylation site is in line with the reported sequence variability of this region among ZIKV strains (Faye et al., 2014) and in other flaviviruses (Sirohi et al., 2016), suggesting that local structural dynamics and sequence variability could be of functional importance for this protein. In fact, sites of various enzymatically-catalyzed posttranslational modifications in proteins are commonly located within their IDPRs (Iakoucheva et al., 2004; Pejaver et al., 2014). Therefore, finding intrinsic disorder in functionally important regions of the ZIKV E protein signifies the potential therapeutic importance of its IDPRs.

### Disorder analysis of the ZIKV non-structural proteins

There are seven non-structural proteins (NS1, NS2A, NS2B, NS3, NS4A, NS4B, and NS5) in ZIKV. Six of the NS proteins (NS2A to NS5) in ZIKV are known to be engaged in the formation of a replication complex on the cytoplasmic side of the endoplasmic reticulum membrane (Song et al., 2016). In addition to the viral non-structural proteins this replication complex also contains several host cofactors (Salonen et al., 2005).

#### NS1 protein

**Figure 5:**
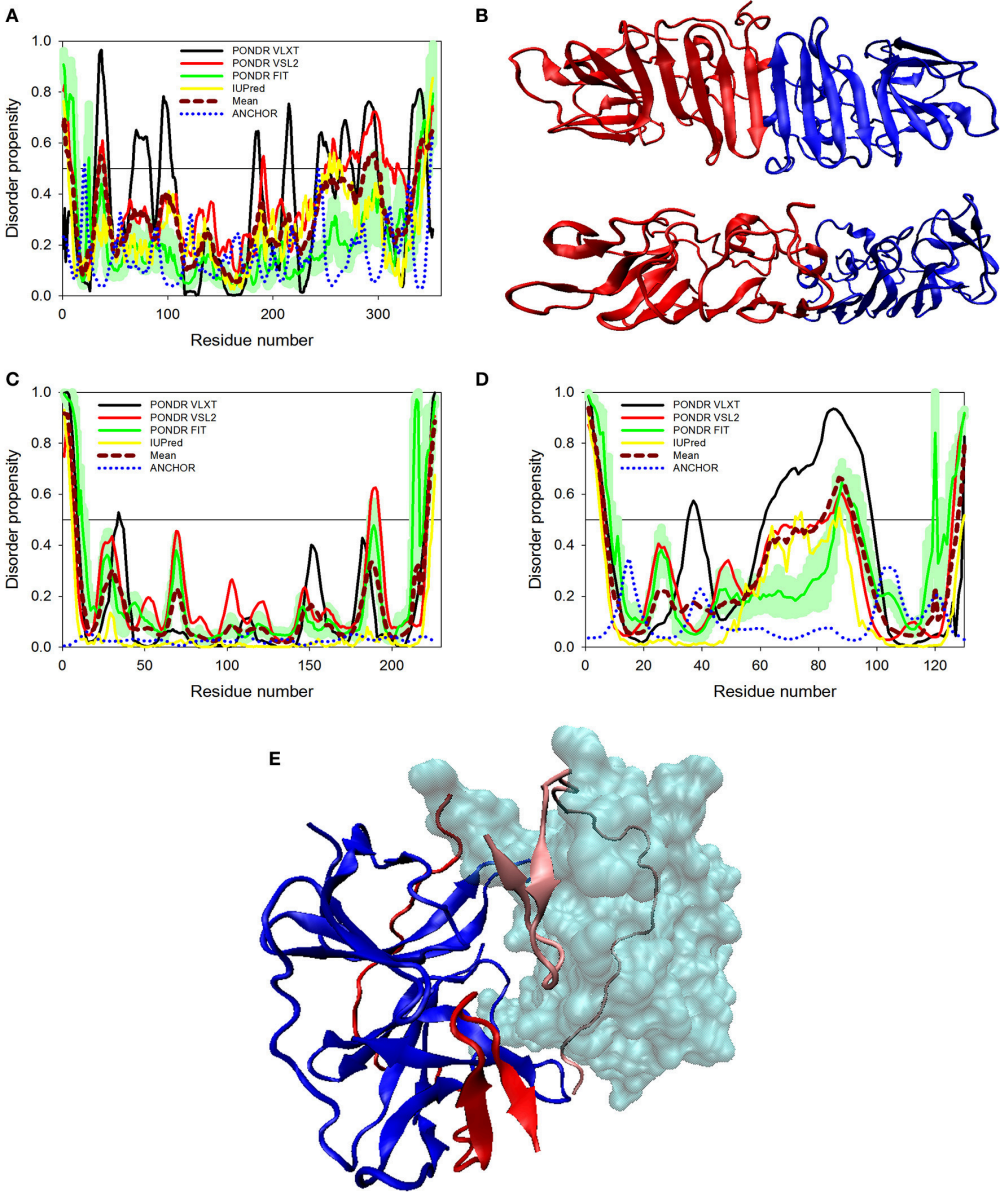
**Disorder predispositions of non-structural proteins NS1 (A)**, NS2A **(C)** and NS2B **(D)**. Disorder profiles generated by PONDR® VLXT, PONDR® VSL2, PONDR® FIT, and IUPred are shown by black, red, green, and yellow lines, respectively. Dark red dashed line shows the mean disorder propensity calculated by averaging disorder profiles of individual predictors. Light green shadow around the PONDR® FIT shows error distribution. Blue dotted lines correspond to the result of the functional disorder analysis using ANCHOR algorithm. **(B)** 3D-structure of the head-to-head dimer of the NS1 protein, where the β-ladder side (top) and loop arrangement (bottom) are shown. Structure is based on the PDB ID: 5IY3 (Song et al., 2016). Structures of monomers within a dimer are shown as blue and red cartoons. **(E)** 3D-structure of a hybrid protein containing NS3-binding region of the NS2B protein covalently linked to the protease domain of ZIKV NS3 protein (PDB ID: 5LC0) (Lei et al., 2016). This hybrid protein is crystallized as a tight dimer. Structures of the NS3-binding region of the NS2B protein are shown as red or pink ribbons in chains **(A,B)** of this dimer, respectively. Structures of the protease domain of NS3 protein are shown as blue ribbon or cyan surface in chains **(A,B)**, respectively. For chain **(B)**, this domain is shown as a transparent surface to simplify visualization of the NS2 chain wrapped around it. Structural representation have been rendered using VMD (Humphrey et al., 1996).

The glycoprotein NS1 is considered as a key molecule in replication, immune evasion and pathogenesis of flaviviruses. NS1 is also secreted out into extracellular space as hexameric lipoprotein particles that are involved in multiple interactions with various components of immune system and host cell molecules (Suthar et al., 2013). In fact, it is believed that NS1 can be responsible for the diverse clinical consequences of infection caused by flaviviruses (Kuno et al., 1998; Cheng et al., 2009). Recently, the crystal structure of a C-terminal fragment of ZIKV NS1 (PDB ID: 5IY3) has been solved (Song et al., 2016). The sequence of this crystallized C-terminal domain (residues 176-351) along with the sequence of the full-length NS1 protein was used in our disorder analysis. This analysis revealed that the full-length NS1 is characterized by the PPID of 10.8% (see Figure 5A and Table 1), whereas the crystallizable C-terminal fragment (PDB ID: 5IY3) shows the PID of 14.1%. Structurally, NS1 is arranged in a rod-like head to head dimers. Each dimer has 20 β-strand showing ladder-like arrangement on one surface and a complex arrangement of several loops on an opposite surface (see Figure 5B) (Song et al., 2016). Typically, loops between the β-strands are short, with the exception to the spaghetti loop connecting strands β4 and β5 (residues 218–272), which lacks regular ordered structure and is predicted to contain significant amount of disorder (see Figure 5A). Furthermore, a potential glycosylation site is located within the short loop between the β3 and β4 strands (residues 207–209), which is also predicted to have high level of disorder. Therefore, the high abundance of disorder in NS1 is correlated with the complex arrangement of loops in 3D crystal structure (Song et al., 2016) (see Figure 5B) and has functional significance. A phylogenetic analysis based on the amino acid sequences of NS1 proteins from 10 flaviviruses revealed some unique sequence characteristics of ZIKV NS1 that positioned it in the individual phylogenetic group (Song et al., 2016). It was emphasized that there is a very large variability in positively and negatively charged surfaces in central loop regions of the NS1 proteins from DENV, WNV, and ZIKV (Song et al., 2016). This variability in loop regions of flaviviruses may be implicated in the diversity of their pathogenicity. In Dengue and WNV, these loops of NS1 have been implicated in immune pathogenesis, whereas in ZIKV, this mechanism needs to be explored (Suthar et al., 2013).

#### Proteins NS2A and NS2B

Non-structural proteins from NS2 to NS5 are involved in the formation of replication complex which is located on the endoplasmic reticulum membrane. NS2A possesses several important functions, such as involvement in viral RNA synthesis, virus-induced membrane formation, and inhibition of interferon α/β response (Xie et al., 2015). Table 1 shows that, being the most ordered of the ZIKV proteins, NS2A has the PPID of 5.4%. However, NS2B is on the other side of spectrum, being characterized by a high abundance of disordered residues with PPID of 16.2 % (Figures 5C,D). Figure 5D shows that NS2B contains a central long disordered region of 37 residues (residues 62-98). This region is responsible for interaction with the NS3 protease (Murray et al., 2008). In the case of DENV it was shown that a central hydrophilic region of NS2B binds to NS3 and is needed for the formation of an active NS2B-NS3 protease complex. NS2B also stabilizes NS3 by acting as a chaperone. In previous studies on other members of the *Flaviviridae* family (Dengue virus and WNV), the structure of NS2B in a complex with NS3 was determined (Erbel et al., 2006). Curiously, recent crystallographic analysis of the hybrid protein constituting a central of the ZIKV NS2B (residues 49–95) covalently linked via the Gly_4_-Ser-Gly_4_ artificial linker to the N-terminal protease domain of NS3 (residues 1–170) revealed that the NS3-binding region of NS2B wraps around the globular NS2B domain (see Figure 5E; PDB ID: 5LC0) (Lei et al., 2016). This structure clearly indicates that this NS2 region is disordered in its unbound form and folds upon binding to the globular protease domain of ZIKV NS3 protein. In the perspective of folding upon binding found for many IDPs, this seems to be a similar mechanism where protein acquires active conformation and function only by binding disordered partner as also evidenced in case of the model IDP systems such as KIX and cMyb (Gianni et al., 2012).

#### NS3 protein

**Figure 6:**
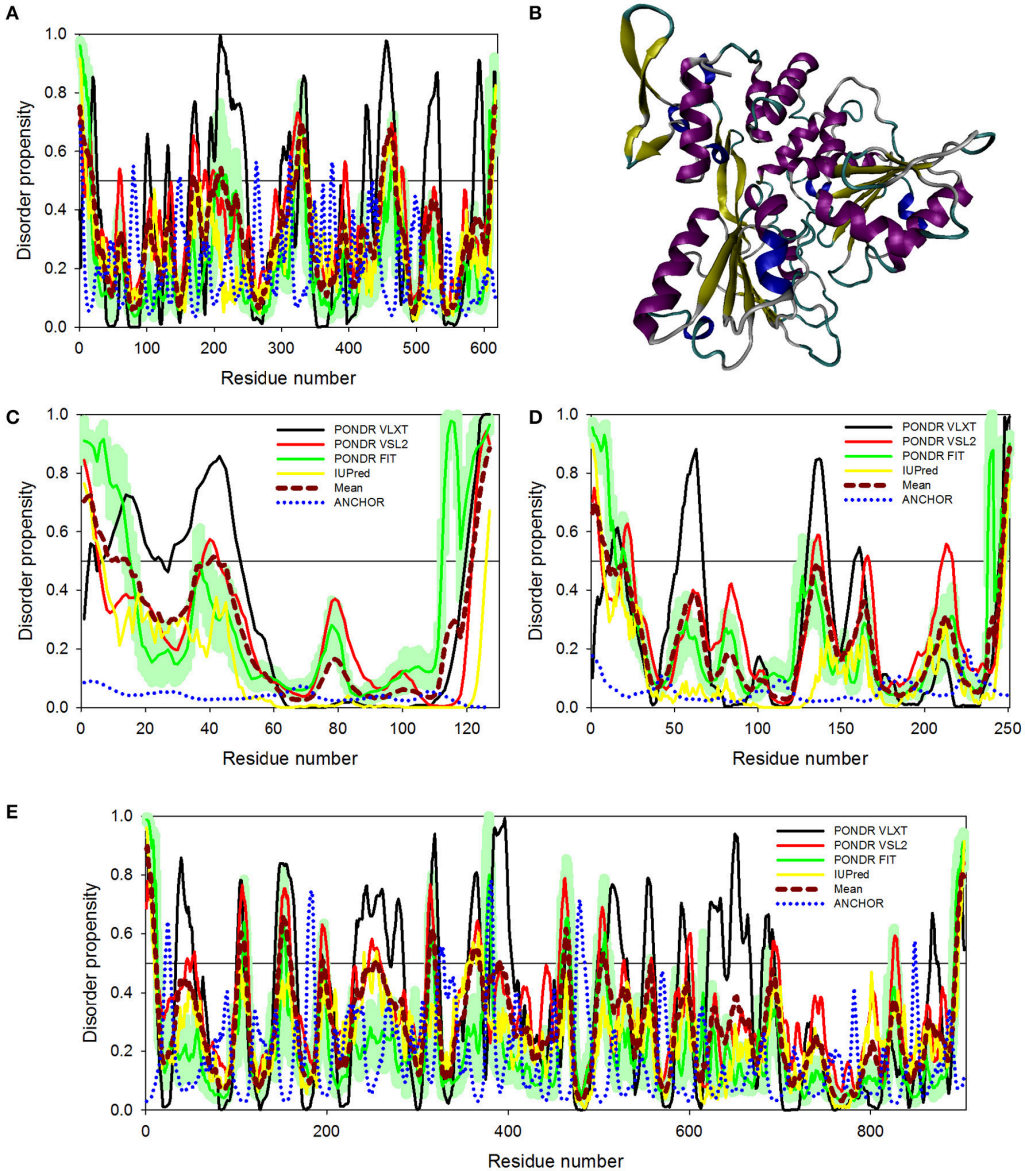
**Disorder predispositions of non-structural proteins NS3 (A)**, NS4A **(C)**, NS4B **(D)**, and NS5 **(E)**. Disorder profiles generated by PONDR® VLXT, PONDR® VSL2, PONDR® FIT, and IUPred are shown by black, red, green, and yellow lines, respectively. Dark red dashed line shows the mean disorder propensity calculated by averaging disorder profiles of individual predictors. Light green shadow around the PONDR® FIT shows error distribution. Blue dotted lines correspond to the result of the functional disorder analysis using ANCHOR algorithm. **(B)** 3D-structure of the helicase domain of the ZIKV NS3 protein (PDB ID: 5JMT) (Tian et al., 2016). Structure is colored according to the secondary structure content. Structural representation have been rendered using VMD (Humphrey et al., 1996).

NS3 is a bifunctional enzyme that consists of two domains, such as the N-terminal protease domain (residues 1–167) and the C-terminal helicase domain (residues 168–617), which are essential for the polyprotein processing and the viral replication, respectively (Luo et al., 2015). NS3 consists of 617 residues, 12.6% of which are predicted to promote disorder. The longest disordered region found in this protein consists of 57 residues (residues 191–247) and is located within the helicase domain (Figure 6A). According to a recent report, Dengue virus NS3 contains the N-terminal proline-rich disordered region that plays a critical role in replication and virus particle formation (Gebhard et al., 2016). As it was already mentioned, the ZIKV NS3 protease domain (residues 1–170) was recently crystallized as a part of the hybrid protein containing the NS3-binding region of the ZIKV NS2B covalently linked by the Gly_4_-Ser-Gly_4_ peptide to N-terminus of the NS3 protein. No structural information was obtained for the 31 residue-long region connecting NS2B and NS3 and containing C-terminal tail of the NS2B, the Gly_4_-Ser-Gly_4_ linker, and the N-terminal 14 residues of the ZIKV NS3 containing the aforementioned proline-rich region (see Figure 5E; PDB ID: 5LC0) (Lei et al., 2016). Remaining part of the NS3 protease domain (except to its last 3 residues) was well-resolved and represents two β-barrels with strand orders AI-BI-CI-αI-DI-EIa-EIb-FI and AII-BIIa-BIIb-CII-DII-EIIa-EIIb-FII (Lei et al., 2016). Recently, a crystal structure of the NS3 helicase domain of ZIKV has been solved (PDB ID: 5JMT) (Tian et al., 2016) (see Figure 6B). In contrast to the DENV NS3 helicase, ZIKV has a monomeric helicase molecule. NS3 helicase is characterized by mostly ordered tertiary structure, with loops serving as connectors between the three domains of this protein (Tian et al., 2016). It was pointed out that the cleft between Domain I and II contains the NTPase active site and includes Walker A (or motif I or P-loop, residues 193–204) and B motifs (or motif II, residues 285–292) that play an important role in recognizing NTP and Mn^2+^ or Mg^2+^ cations (Caruthers and McKay, 2002; Tian et al., 2016). Figure 6A shows that both of these motifs are located within IDPRs. Although these loops are characterized by the considerable intrinsic flexibility, it was pointed out that they are highly conserved among flaviviruses and play an important role in binding and catalysis of NTP (Tian et al., 2016). Curiously, NS3 is one of the two ZIKV proteins that are predicted to have some disorder-based binding sites identified by ANCHOR. In fact, Figure 6A shows that there are 6 such sites in ZIKV NS3 (residues 1–4, 79–81, 147–150, 261–264, 311–314, and 374–376) which although were predicted by ANCHOR but then were filtered out by the algorithm because of their small size (they are ranging in length between 3 and 4 residues and ANCHOR uses the length threshold of 6 residues). Nevertheless, our analysis shows that ZIKV NS3 has several functionally important IDPRs.

#### NS4A and NS4B proteins

Although non-structural proteins NS4A and NS4B from flaviviruses have not been crystallized as of yet, their biological roles in Dengue virus have been investigated experimentally. (Zou et al., 2015) NS4A functions by introducing rearrangements in membrane of host endoplasmic reticulum. These changes lead to the formation of virus-induced membranous vesicles. NS4A also control the ATPase activity of the NS3 helicase, whereas NS4B inhibits the interferon-induced host STAT1 phosphorylation and nuclear translocation (Kuno and Chang, 2007). Table 1 and Figures 6C,D show that although NS4A is predicted to be rather disordered, having the PPID of 16.5%, the level of intrinsic disorder in the NS4B protein is noticeably lower (its PPID is of 6.4%).

#### NS5 protein

The NS5 protein is the largest ZIKV protein (903 residues) and has high degree of sequence similarity with the Dengue virus NS5 protein. In DENV, crystal structure of the full-length NS5 has been solved recently (PDB ID: 4V0Q) (Zhao et al., 2015), and structures of the methyltransferase domain (PDB ID: 1L9K) (Egloff et al., 2002), and the RNA-dependent RNA polymerase (RdRp) domain (PDB ID: 2J7W) of the NS5 (Yap et al., 2007) are also available. The NS5 is the most conserved protein amongst flaviviruses, exhibiting enzymatic activities that play vital roles in virus replication. In fact, it has the N-terminal domain (residues 1–262 in DENV3) that belongs to the S-adenosyl-L-methionine (SAM)-dependent methyltransferase (MTase) superfamily (Egloff et al., 2002) and the C-terminal domain (residues 273–900) which serves as the RNA-dependent RNA polymerase (RdRp) that synthesizes the anti-genome and progeny genome (Yap et al., 2007). Since NS5 contains two functional domains with several key enzymatic activities crucial for the viral RNA replication in the host cell, this protein represents one of the major targets for the design of antiviral inhibitors. Our analysis revealed that despite being the multifunctional enzyme, ZIKV NS5 contains numerous IDPRs (in a range of 20) and is characterized by the PPID of 8.6%. Furthermore, ANCHOR analysis revealed the presence of 4 disorder-based binding sites (residues 22–27, 179–186, 324–329, and 377–383), with two more disorder-based binding sites that were filtered out by the algorithm (residues 476–484 and 846–849). Our analysis also revealed that most of the NS5 disordered regions are located in the central part of the protein, with the longest disordered region containing 40 amino acid residues (see Figure 6E). These findings of NS5 are also correlating well with the presence of long disordered region in DENV and JEV NS5 proteins.

## Concluding remarks

In conclusion, our analysis revealed that all ZIKV proteins contain disordered regions. These proteins are involved in diverse mechanisms of virus survival and immune evasion in other flaviviruses. Till now, the mechanisms of the ZIKV pathogenesis have not been deciphered in detail. Therefore, our study that considers ZIKV proteins from the protein intrinsic disorder perspective may provide novel insights that can help elucidating the molecular mechanisms of virus host interaction. In general, the highly dynamic and flexible nature of the disordered proteins or proteins containing IDPRs has shown to play major roles in disease development and progression (Uversky et al., 2008; Babu et al., 2011). As disordered regions are attractive and challenging drug targets, new drug development strategies should be developed to find small molecule inhibitors that could target IDPRs and could serve as effective antivirals. Therefore, detailed biophysical analysis of ZIKV disordered proteins is required to develop effective new antiviral therapeutics.

## Author contributions

RG and VU: Conception and design, analysis and interpretation of data, writing and review of the manuscript and study supervision; DK and NS: acquisition of data, analysis and interpretation of data, writing of the manuscript.

## Funding

This work was partially supported by DST grant, India (YSS/2015/000613) to RG and IIT-Mandi, India to RG. DK is supported by ICMR fellowship and NS is supported by MHRD fellowship, both in India.

### Conflict of interest statement

The authors declare that the research was conducted in the absence of any commercial or financial relationships that could be construed as a potential conflict of interest. The reviewer NF and handling Editor declared their shared affiliation, and the handling Editor states that the process nevertheless met the standards of a fair and objective review.
